# 

**Published:** 1899-10

**Authors:** 


### MARRIAGE.

AT 419 Elmwood Avenue, Buffalo, N. Y., on October 11, 1899, Dr. Ernest L. Volgenau, of the Bureau of Animal Industry staff, and now stationed at New Haven, Conn., to Miss Jessie Katharine Bernhardt, of the former city. The happy couple will make their future home in the "City of Elms."

---

The souvenir number of the *Horseshoers' Journal* is a production worthy of so important a craft, and reflects much credit on the editorship of the *Journal*. It is replete with valuable information to the craft.

### PERSONALS.

Dr. Charles E. Cotton, of Minneapolis, Minn., concluded his Eastern visit by a stay of several days among his old classmates in and around Philadelphia.

Dr. J. C. Bartholomew, of Berwyn, Pa., was an exhibitor at the Bryn Mawr horseshow in September.

Dr. E. Mayhew Michener, of North Wales, Pa., is the happy possessor of a daughter, a recent arrival in the family.

Dr. George Lytle, of the Bureau of Animal Industry of Chicago, has recently taken unto himself a wife.

Dr. T. Bent Cotton, of Mt. Vernon, Ohio, was a stroller along Philadelphia's business streets before returning to his home.

Dr. George W. Turner, Veterinarian, U. S. Army, has been transferred from his former station, Chattanooga, Tenn., to Fort Sheridan, Ill.

Dr. William E. Howe, formerly of Syracuse, N. Y., and more recently an attache of the Bureau of Animal Industry of Chicago, has tired of single-blessedness and become a benedict.

Dr. M. J. Henderson, of Syracuse, N. Y., is a fancier of the horse of speed, and will enter the arena of trotters this fall with a very fast stallion.

Dr. John M. Parker, of Haverhill, Mass., has just returned from a two months' trip abroad, very much improved in health, and after a very enjoyable absence from routine duties.

Lieutenant-Colonel R. S. Huidekoper, of the First Army Corps, at the Convention of Military Suregons in Kansas City, Mo., in September, gave a serious review of the blunders of the Surgeon-General's office in our late war, and laid much of the fault at the door of Surgeon-General Sternberg. His paper was an ably prepared one, and will shed much light on the army department's shortcomings in the care and protection of the soldiers from a medical point of view. It is a serious answer to the jackal newspapers that attacked Dr. Huidekoper when in a position where defence could not be established without violating army regulations.

Drs. Andrew Darling and J. M. Phillips will officiate as veterinary inspectors this year at the St. Louis horse show the latter part of October and the first of November.

